# Ensiling of crops for biogas production: effects on methane yield and total solids determination

**DOI:** 10.1186/1754-6834-4-44

**Published:** 2011-10-27

**Authors:** Emma Kreuger, Ivo Achu Nges, Lovisa Björnsson

**Affiliations:** 1 Department of Biotechnology, Lund University, PO Box 124, SE-22100, Lund, Sweden

**Keywords:** biogas, anaerobic digestion, methane potential, biofuel, ethanol, volatile fatty acids, dry matter, total solids, volatile solids, ensiling, silage

## Abstract

**Background:**

Ensiling is a common method of preserving energy crops for anaerobic digestion, and many scientific studies report that ensiling increases the methane yield. In this study, the ensiling process and the methane yields before and after ensiling were studied for four crop materials.

**Results:**

The changes in wet weight and total solids (TS) during ensiling were small and the loss of energy negligible. The methane yields related to wet weight and to volatile solids (VS) were not significantly different before and after ensiling when the VS were corrected for loss of volatile compounds during TS and VS determination. However, when the TS were measured according to standard methods and not corrected for losses of volatile compounds, the TS loss during ensiling was overestimated for maize and sugar beet. The same methodological error leads to overestimation of methane yields; when TS and VS were not corrected the methane yield appeared to be 51% higher for ensiled than fresh sugar beet.

**Conclusions:**

Ensiling did not increase the methane yield of the studied crops. Published methane yields, as well as other information on silage related to uncorrected amounts of TS and VS, should be regarded with caution.

## Background

Biogas production using energy crops as the main feedstock is attracting increasing attention. Germany is leading the field, with almost 3, 900 biogas plants in operation in 2009, the majority using ensiled crops [1]. Ensiling is a traditional method of preserving animal feed, and can also be used to store crops intended for biogas production [2]. The amounts of total solids (TS) or dry matter (DM) and volatile solids (VS) are often used to characterize the ensiled material added to the biogas process, and to calculate the methane yield from the material. A standard method of determining the TS of biomass is oven drying at 105°C [3,4]. Other oven temperatures, such as 60°C, 85°C or 100°C are also common [3,5]. In this paper total solids (TS) and dry matter (DM) are regarded as being equivalent, and the term used is that used in the publications referred to.

At the beginning of the 20th century it was reported that oven drying gives inaccurate values of the DM when the sample contains volatile compounds. It should therefore not be applied to silage as it contains varying amounts of volatile fatty acids (VFAs), lactic acid, ammonia and alcohols formed during the ensiling process [6,7]. McDonald and Dewar [8] quantified the losses of volatile compounds during oven drying by condensing and analyzing the vapor. A year later, they described a method in which the water content was determined through toluene distillation, with corrections for organic acids, ethanol and ammonia in the distillate [9]. The corrected toluene extraction method was long used as a standard method for determining the DM in silage used for fodder production, but was abandoned due to the harmful nature of toluene. The most common method used today to determine the DM in silage is oven drying, with corrections for the volatilization of VFAs, lactic acid, alcohols and ammonia. The type and amount of volatile compounds lost depends on the drying temperature, and different coefficients are used to adjust the DM for the expected losses of individual compounds at certain drying temperatures [5,10]. The adjusted values are referred to as corrected DM or corrected TS.

Although the limitations of using oven drying without correction for volatile compounds have been known for many years in agricultural sciences, the method is still routinely used in research related to methane production through anaerobic digestion. The methane yield from anaerobic digestion is normally expressed per unit of VS. The amount of VS is based on the amount of TS, which is determined according to standard methods by oven drying, without correction for volatile compounds [4]. After oven drying, the dry material is incinerated at 550°C to determine the ash content. The difference between the TS and the ash is defined as the VS. This means that if the TS are underestimated the VS will also be underestimated. If the VS of the silage are underestimated, the loss of VS during ensiling will be overestimated, and the methane yield per unit VS will be overestimated.

VS losses of 18% to 35% due to ensiling have been reported [11]. At the same time, ensiling has been reported to increase the methane yield of the material by 25% to 42% [11,12]. Results such as these may be the result of losses of volatile compounds during VS determination. There are several other recent examples of this, where the methane yields reported from ensiled grass, maize and beet were based on methods of TS or VS determination without correction for the loss of volatile compounds (see, for example, [13-17]). The VS-based methane yields given for ensiled materials may therefore be overestimated. Yields from silage based on uncorrected TS and VS values have been reported in other biofuel fields as well, such as ethanol research [18,19].

Although no biogas-related research has, until very recently [20], made use of the thorough internationally published studies performed on silage for fodder, some authors have considered the fact that volatile compounds may be lost during the determination of TS and VS. It is mentioned in the standard method of the American Public Health Association (APHA) [4] that losses of volatile organic matter from the sample can cause a negative error, but no further comments are made on how this error can be corrected. Angelidaki *et al. *[21] suggest drying at a lower temperature (90°C) after increasing the pH of the sample. However, according to Porter and Murray [5], neither drying at lower temperature nor increasing the pH decreased the volatilization of alcohols. Demirel and Scherer [22] described a method of VS determination applied to beet silage, in which suspended solids and dissolved solids (VFAs, lactic acid and alcohols) were analyzed separately, by drying and gas chromatography, respectively, and then combined to give the total VS. However, dissolved organic compounds other than VFAs, lactic acid and alcohols will not be included. Methods, including volatilization coefficients, have been presented in publications by Weissbach and Strubelt [23-26] and Mukengele and Oechsner [27] in a German journal for agricultural technology. Volatilization coefficients for correcting oven-dry-based DM for ensiled crops are outlined, and the methods described are similar to that presented by Porter and Murray [5]. Unfortunately, these articles will not be found via scientific search engines such as ISI Web of Science, Scifinder and SciVerse ScienceDirect, and the articles refer to methods published in German (see, for example, [28]). Two recent publications [20,29] concerning the influence of ensiling on the methane potential do make use of correction factors [10,28]. However, none of them emphasize the importance of correcting TS and VS, to avoid overestimating methane yields, and both refer to previously published results based on uncorrected TS and VS without comment or concern about the reliability.

Among others, McDonald *et al. *[30] have pointed out that, even when using corrected DM, the change in DM during ensiling does not provide a measure of the change in the energy content of the silage, since the two are not correlated (as can be seen in Table 1). The fermentation of sugar to acetic acid or lactic acid will not influence the potential for methane production (Table 1). Fermentation to ethanol results in the concentration of the energy in the dry matter, and part of the dry matter is lost as carbon dioxide, while most of the energy is retained in the product (Table 1). The stoichiometric methane potential of glucose, acetic acid and lactic acid is 374 l/kg VS and, for the more reduced carbon source ethanol it is 731 l/kg VS. Only in cases of undesirable fermentation, such as butyrate fermentation, is a considerable amount of energy truly lost due to the release of hydrogen (see Table 1). In well preserved silage, the butyrate concentration is low [30].

**Table 1:** Mass and energy recovery for fermentation during ensiling

Type of fermentation	Product	Mass recovery	Energy recovery
Homolactic fermentation	2C_3_H_6_O_3_	100%	97%

Acetic acid fermentation	3C_2_H_4_O_2_	100%	93%

Heterolactic fermentation	C_3_H_6_O_3 _+ C_2_H_6_O + CO_2_	76%	97%

Ethanol fermentation	2C_2_H_6_O + 2CO_2_	51%	97%

Butyrate fermentation^a^	C_4_H_8_O_2 _+ 2CO_2 _+ 2H_2_	49%	78%

Mass and energy recovery for some common fermentation pathways during ensiling [30]. The examples are based on glucose as substrate. Gases are regarded as lost. Energy recovery is based on the gross energy value (higher heating value) of the products, excluding the energy in ATP.

^a^Performed by many *Clostridia *species.

The purpose of the current study was to examine how ensiling influences the methane potential, the mass and the total solids of crops. Furthermore, we wished to draw attention to the errors that can arise from using uncorrected, oven-dry-based values of TS and VS, and to highlight a previously presented method, for correcting oven-dry-based TS and VS values for losses of volatile fermentation products during oven drying [5]. The method developed for grass silage was tested on four other crop materials. Laboratory-scale ensiling was performed, followed by methane production from ensiled and non-ensiled crops. The losses in wet weight, and the production of methane and hydrogen and total gas volume during ensiling were determined. The content of the dominating volatile organic compounds in silage were measured before and after standard TS determination of the ensiled crops and used to calculate corrected TS and VS contents. The TS and VS contents were corrected in two ways: one using the volatilization coefficients presented by Porter and Murray [5], and the other (for validation) by adding the fraction of volatile compounds lost during drying. The volatilization coefficients from Porter and Murray [5] were used since they are based on silages mainly prepared with bacterial inoculants [5] rather than silages prepared with formic acid [10]. Four crop materials were chosen for the study: maize, which is the dominating crop used for anaerobic digestion in Europe; hemp, which is more fibrous than maize; and sugar beet (beets and beet tops ensiled separately), which contain less fiber and more soluble sugars than maize.

## Results and Discussion

### Comparison of the changes in wet weight, TS and VS during ensiling based on uncorrected and corrected values

The wet weight was found to decrease during ensiling by about 1% for all materials except beets, for which the decrease was about 4% (Table 2). For sugar beets and maize, the decrease in TS during ensiling was significantly higher than the decrease in wet weight when using the uncorrected TS content, demonstrating the error in the method (rows E and F in Table 2). After correcting the TS contents of the silages the decrease in TS (row K, Table 2) was no longer larger than the decrease in wet weight for any of the materials.

**Table 2:** Changes in wet weight (WW) and total solids (TS) during ensiling

Row		Percentage of	Maize	Hemp	Beets	Beet tops
A	Ensiling replicates, n		4	2	3	4

B	TS prior to ensiling^a^	Fresh WW	26.8 ± 0.2	31.4 ± 2.1	23.0 ± 0.2	13.2 ± 1.6

C	VS prior to ensiling^a^	Fresh WW	25.0 ± 0.1	28.4 ± 0.4	21.3 ± 0.9	10.6 ± 0.6

D	Uncorrected TS after ensiling^b^	Silage WW	24.5 ± 0.8	29.4 ± 0.4	14.2 ± 0.1	10.4 ± 0.4

E	Weight after ensiling	Fresh WW	99.2 ± 0.0	98.4 ± 0.1	95.6 ± 0.3	99.0 ± 0.5

F	Decrease in TS based on uncorrected TS^c^	Fresh WW	2.5 ± 0.8	2.4 ± 2.1	9.5 ± 0.2	2.9 ± 1.6

G	Maximum CO_2 _relased^d^	Fresh WW	0.5	1.5	3.3	0.7

H	TS after ensiling based on CO_2 _release^e^	Silage WW	26.5	30.4	20.6	12.6

I	Corrected TS after ensiling according to Porter and Murray^f^	Silage WW	26.4 ± 0.1	30.7 ± 0.5	23.3 ± 1.1	13.1 ± 0.7

J	Corrected TS after ensiling based on measurements^g^	Silage WW	26.5 ± 0.1	30.4 ± 0.5	23.8 ± 1.1	13.6 ± 0.7

K	Decrease in TS, corrected according to Porter and Murray [5]^h^	Fresh WW	0.6 ± 0.2	1.2 ± 2.2	0.7 ± 1.0	0.2 ± 1.8

Changes in W and TS during ensiling, expressed as percentage of fresh crop or silage WW (mean ± SD). TS content was determined in duplicate. Decrease in WW and the maximum amount of CO_2 _released were determined for the number of ensiling replicates given in row A.

^a^Measured on fresh crops with ensiling solution.

^b^The TS content was analysed for both ensiled crops directly after opening the buckets (the value given here) and after freezing (the value used for correcting TS and VS, since VFAs, lactic acid and alcohols were determined after freezing). No significant difference was seen between the two measurements.

^c^Calculated according to: B - D × (E/100) (letters indicate rows).

^d^Based on the total amount of gas released and the estimated amount of CO_2 _in the ensiling buckets minus methane, and hydrogen and the estimated amount of nitrogen gas in the buckets at the start of ensiling.

^e^Calculated according to: (B - G)/(E/100) (letters indicate rows).

^f^TS values in row D plus 37.5% of the lactic acid, 100% of the ethanol and 89.2% of the acetic and butyric acid present in the silage (Table 3), according to Porter and Murray [5].

^g^TS values in row D plus the difference between the contents of lactic acid, ethanol, acetic acid and butyric acid in the ensiled crops before and after TS determination.

^h^Calculated according to: B - I × (E/100) (letters indicate rows).

TS, total solids; VFA, volatile fatty acid; VS, volatile solids; WW, wet weight.

Ethanol and acetic acid were present in all silages (Table 3). Lactic acid was present in all silages except the hemp silage (Table 3). Butyric acid (Table 3) and very small amounts of propionic and succinic acid (less than 0.1% of the wet weight) were detected in hemp silage, but not in the other silages. The pH of the hemp silage was higher than the other silages; namely 4.5, compared with 3.1 for maize, 3.0 for beet tops and 2.9 for beets.

**Table 3:** Volatile compounds in ensiled crops

Substrate	n	Lactic acid	Ethanol	Acetic acid	Butyric acid	Total
Maize	2	1.26 ± 0.02	0.21 ± 0.00	0.74 ± 0.04	BD	2.21 ± 0.05

Hemp	2	BD	0.29 ± 0.01	0.94 ± 0.04	0.11 ± 0.01	1.13 ± 0.04

Beets	2	0.91 ± 0.07	4.82 ± 0.86	1.09 ± 0.14	BD	6.82 ± 0.87

Beet tops	2	1.08 ± 0.04	0.53 ± 0.04	0.56 ± 0.00	BD	2.18 ± 0.06

Contents of volatile compounds measured in the ensiled crops, expressed as percentage of wet weight (mean ± SD). Determinations were made in duplicate starting with the steeping step.

BD, below detection limit.

After drying the silages no ethanol could be detected, and lactic, acetic and butyric acid were found at lower concentrations. On average, 100% (± 0%) of the ethanol (n = 8), 53% (± 13%) of the lactic acid (n = 6), 72% (± 0.01) of the butyric acid (n = 2) and 89% (± 17%) of the acetic acid (n = 8) evaporated during TS determination. The average values are not significantly different from those presented by Porter and Murray [5]: 97.5% for ethanol, 37.5% for lactic acid and 89.2% for acetic and butyric acid. However, there is considerable variation in volatilization between the samples as indicated by the SDs, showing that there is room for further improvement of the method. The volatilization coefficients used by Weissbach and Strubelt [25], included a pH dependency for the VFAs, which may further increase the accuracy of the corrected values. The volatilization coefficients presented in that article cannot be compared to those obtained here since they used different drying conditions (initial drying at 60°C, followed by drying at 105°C) from those used in this study (105°C).

Corrected TS contents are presented in rows I and J in Table 2. The values in row I are calculated based on the concentrations in the silages and the volatilization coefficients given by Porter and Murray [5]. The values in row J are based on the experimentally determined volatilization during oven drying, that is, the difference between the content of volatiles before (Table 2) and after (data not shown) TS determination by oven drying. No significant differences were found between the results obtained with the two methods, showing that the volatilization coefficients presented by Porter and Murray [5] give good estimates of the true TS for the silages investigated. Theoretical calculations of the TS contents after ensiling, based on the gas production and weight changes (row H, Table 2), gave values in line with those obtained with corrections for losses of volatiles (rows I and J, Table 2).

### Gas production and energy losses during ensiling

The production of energy-containing gases such as hydrogen and methane during ensiling was negligible in all cases: less than 0.1 ml per g VS for all substrates except hemp, which gave less than 2 ml hydrogen per g VS. The energy contained in the hydrogen produced by hemp during ensiling corresponded to about 2 ppm of the energy in the methane produced in the biochemical methane potential (BMP) test. For hemp, beets and beet tops, only hydrogen and no methane was detected; for maize, methane but no hydrogen was detected. The low production of energy-containing gases, together with the low pH in all the silages, except hemp, indicates that the silages were well preserved.

For maize, hemp and beet tops, 67% to 89% of the total gas produced (including carbon dioxide) during ensiling was produced during the first 4 days. The gas produced by beet silage was higher than that produced by the other crops, with high gas production during the first 4 days, and a second gas production peak around days 9 to 13, giving 72% of the total gas production between days 6 and 17. All crops produced less than 6% of the total gas between days 30 and 60. After 60 days, the buckets were moved from storage at room temperature to 4°C. Very little gas was produced after this, less than 1% by all crops except hemp, which produced around 5% of the total gas during this time.

The maximum mass loss due to aerobic degradation resulting from entrapped oxygen at the start of the ensiling process was calculated and found to be negligible, at most 0.025% of the wet weight. The calculation was based on the assumption that the maximum volume of entrapped air was the volume of the bucket minus the volume of the substrate at the start of ensiling (assuming a density of the substrate of 1 kg/l), 21% of the air being oxygen.

### BMP tests

**Figure 1:**
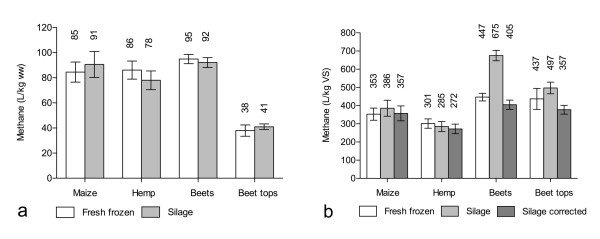
**Methane yields of fresh frozen and ensiled crops**. Methane yields expressed as **(a) **l per kg wet weight (WW) (left), and **(b) **l per kg volatile solids (VS) (right). The methane yields are given above the bars. Error bars denote 1 SD, n = 3.

The methane potential was determined and is expressed per unit wet weight (Figure 1a) and per unit uncorrected and corrected VS for silages (Figure 1b). When expressing the methane yield per unit wet weight (Figure 1a) or per unit VS corrected according to Porter and Murray [5] (Figure 1b) no significant difference was seen between fresh frozen and ensiled material for any of the crops. Neither was there any significant difference between the methane yields from fresh frozen crops and ensiled crops related to the wet weight or VS of the original materials (taking mass losses during ensiling into account).

When relating the methane yield from ensiled material to uncorrected VS, the results are noticeably different. The apparent methane yield from beets was significantly higher (51%) from ensiled material than from fresh frozen material when expressing the yield per unit uncorrected VS (Figure 1b). A significant difference was also seen between the methane yield from silage expressed per unit uncorrected and corrected VS for beets and beet tops (Figure 1b).

Herrmann *et al. *[29] found that the methane yields were significantly higher after ensiling in 44% of the cases investigated, when the methane yields of the silages were related to the corrected VS of the silages, but not when they were related to the original VS. Pakarinen *et al. *[20] found methane yields after ensiling to be everything from unchanged to decreasing or increasing compared to yields from fresh crops. Pakarinen *et al. *[20] did not relate their results to original VS since changes in TS and VS during ensiling were not recorded.

The overestimated methane yield of beet silage and beet top silage in the current study, and the fact that the TS losses appeared higher than the wet weight losses for beets and maize when using uncorrected TS and VS contents, demonstrate that methane yields of silages based on uncorrected TS and VS are unreliable.

## Conclusions

Ensiling was not found to increase the methane yield from any of the crop materials investigated in this study. Instead, it was shown that observations such as increased VS-based methane yields or TS losses during ensiling may be artifacts caused by errors in the standard methods commonly used for TS and VS determination. Oven-dry-based TS and VS determination without correction for the loss of volatile compounds is an unsuitable method for all substrates containing noteworthy amounts of volatile compounds. This applies to ensiled energy crops as well as other materials, and is important when using the substrate for anaerobic digestion as well as for other purposes. Caution should therefore be exercised when considering published information about silages, and other materials containing volatile compounds, based on TS and VS. The application of a method developed for grass silage for correcting TS and VS [5], to other ensiled crops, eliminated the significant error of using uncorrected TS and VS. However, the method can be improved further.

## Methods

### Crops

Hemp (Futura 75), maize (Arabica) and sugar beet (EB 726 (Syngenta, Basel, Switzerland), a non-commercially available cultivar with lower sugar content and higher biomass yield than normal sugar beet) were cultivated in southern Sweden (Lönnstorp, Lomma, 55 40'N 13 6'E). The crops were harvested on the following dates: hemp on 5 September 2007, maize on 29 September 2008, and sugar beet on 21 October 2008. Hemp and sugar beet were harvested manually. Maize was harvested with a maize forager set at a chopping length of 10 mm. The hemp and sugar beet tops (leaves and the neck of the root) were chopped in a garden shredder (AXT 2500 HT, Robert Bosch GmbH, Germany) into pieces about 2 cm long. The sugar beets were cut into 1 cm slices and then into squares measuring 2 to 3 cm. Part of each crop material was ensiled directly and part was frozen for later analysis. The TS and VS contents were determined in fresh crops before ensiling with and without ensiling inoculant, in fresh crops after freezing, and in ensiled crops before and after freezing. TS corrected for volatile compounds were determined in frozen ensiled crops. (Frozen samples were used since the authors were not aware of the corrected method prior to freezing the silage.)

### Ensiling

Ensiling was carried out in 4.8 l plastic buckets with tightly fitting lids, normally used for food storage (NordicPack, Nykvarn, Sweden). Hemp, maize, sugar beets (beets) and sugar beet leaves including the upper part of the roots (beet tops) were ensiled separately, using four replicate buckets for each kind of crop material. A gas collection system was made by connecting Tygon tubing (VWR International, West Chester, PA, USA) to a balloon made of Transfoil El-OPET/PE (Flextrus AB, Lund, Sweden) with a hose connector (Slangservice i Uppsala AB, Uppsala, Sweden) in each lid. Silicone was used to seal the connection between the hose connector and the lid and between the bucket and the lid. The chopped plant material was sprayed with a bacterial ensiling inoculant, Lactisil Stabil (Chr. Hansen A/S, Hørsholm, Denmark). In all, 20 ml was added per kg wet plant material, according to the manufacturer's instructions (1.25 g powder in 5 l tap water). The decrease in weight was recorded by weighing the material in the buckets before and after the ensiling period. The decrease in TS was determined based on the wet weight and TS of the fresh crops with ensiling solution and of ensiled crops.

The buckets were stored at room temperature (23 to 25°C) for 60 days; after which they were stored at 4°C for a minimum of 100 days. The gas volume and the contents of methane and hydrogen were monitored during the entire ensiling period. The results from one bucket of beets and two buckets of hemp were excluded due to gas leakage.

The replicate samples of each crop material were mixed after ensiling before sampling for TS and VS determination, and for BMP tests. The mixed samples were also frozen for later analyses. TS determination and BMP tests were started immediately after sampling to minimize losses due to volatilization during sample handling. Contents of VFAs, lactic acid and ethanol were determined in silage samples that had been frozen, since this part of the study was included later. Prior to analysis, frozen silages were thawed at 8°C in buckets with tightly fitting lids.

### BMP tests

BMP tests were performed as reported elsewhere [31], with the modifications described below. Fresh frozen crops, ensiled crops (not frozen) and control samples (described below) were tested in triplicate. The inoculum-to-sample ratio was 2:1 in terms of VS of the fresh frozen crops; silage was added based on the same wet weight as the fresh frozen crops. A total of 300 ml of inoculum was added to each test flask. Inoculum was collected from an anaerobic codigestion plant (Söderåsens Bioenergi, Wrams Gunnarstorp, Sweden). This inoculum is rich in macronutrients and also contains relatively high amounts of trace elements, therefore no nutrients were added. The reaction temperature was set to 38°C. The inoculum was preincubated at 38°C for 5 days prior to the start of the experiment.

The total gas volume and the content of methane [31] were monitored every day for the first week, and then every third or fourth day thereafter, until the end of the experiment. Two sets of controls were included: one set in which only the inoculum was used (to measure the indigenous methane production from the inoculum, which was subtracted from the total methane produced), and a second with microcrystalline cellulose (Avicel PH-101, Sigma-Aldrich, St. Louis, MO, USA) to test the activity of the inoculum. The experiments were terminated after 30 days. The methane yield was related to the wet weight and to the TS and VS of fresh substrate with ensiling inoculant and ensiled substrate. For ensiled substrates the methane yields were also related to the VS content corrected according to Porter and Murray [5]; VS contents determined after freezing were used for this since these were the materials used for determination of the volatile compounds.

### Analyses

TS and VS were determined in duplicate or quadruplicate according to standard methods [4], using samples of 13 to 240 g instead of 25 to 50 g. The TS of each substrate were measured several times, for example before and after the addition of ensiling solution, before and after freezing, and so on. In each case, the TS value corresponding to the actual material used was used for calculations. Corrected values of TS and VS were determined similarly to those presented by Porter and Murray [5]. Duplicate samples of 60 g thawed frozen silage (mixture of material from all ensiling replicates) were steeped in 300 g deionized water for 15 to 19 h at 8°C in a 500 ml flask with a lid. For beets and beet tops the material was separated into a solid and a liquid part (6% liquid for beets and 15% for beet tops) before sampling. The pH was measured after steeping and the pH of undiluted silage was calculated. Quadruplicate samples of the same material were analyzed by drying 13 to 41 g wet weight in aluminum crucibles at 100 to 105°C for 20 to 24 h, according to standard methods to determine TS [4]. Two of the quadruplicates of the dried samples were steeped in deionized water in the same proportions as for the wet silage (1:5), and the other two samples were used for VS determination according to standard methods. Steeping was performed in crucibles covered with several layers of Parafilm. Liquid samples were acidified with H_2_SO_4 _to a pH of 1 to 3 and filtrated through 0.45 μm polypropylene filters (Chromacol, Welwyn Garden City, UK). The content of C_1_-C_6 _VFAs (including isoforms of butyric and valeric acid), lactic acid, succinic acid and ethanol were determined using high performance liquid chromatography (HPLC) (Jasco Co., Tokyo, Japan) with an Aminex HPX-87H column (Bio-Rad Laboratories Inc., Hercules, CA, USA) and a refractive index detector (Erc Inc., Huntsville, AL, USA). Sulfuric acid (5 mM) was used as the mobile phase (0.6 ml/min), and the oven temperature was 40°C. The concentration of VFAs, lactic acid and ethanol and were calculated for the wet silage according to Equations 1 and 2:

(1) $$\text{Concentration in wet silage }\left(\text{g}∕\text{kg}\right)=\left({\text{m}}_{\text{1}}+{\text{m}}_{2}-{\text{m}}_{3}\right)\times {\text{c}}_{\text{1}}∕{\text{m}}_{\text{1}}$$

(2) $$\text{Concentration after drying related to wet silage }\left(\text{g}∕\text{kg}\right)\;={\text{c}}_{\text{1}}\;\times \text{ D }\times {\text{m}}_{3}∕{\text{m}}_{\text{1}}$$

Where m_1 _= original wet weight related to TS added, g; m_2 _= water added, g; m_3 _= substrate TS added, g; c_1 _= concentration of analyzed compound, g/kg; and D = dilution factor = 5.

The TS and VS were corrected in two ways: (1) according to the volatilization coefficients for grass silage dried at 100°C presented by Porter and Murray [5]: lactic acid 0.375, total VFAs 0.892 and ethanol 1.000; and (2) the measured losses of VFAs, ethanol and lactic acid during drying (the difference between Equations 1 and 2) were added to the TS and VS values measured using standard methods.

Gas composition with respect to methane was determined using gas chromatography and a thermal conductivity detector, as described elsewhere [32]. Hydrogen was analyzed in an identical system but with argon as the carrier gas. The gas volume was measured using a graduated 100 ml gas-tight glass syringe (Fortuna, Germany) with a sample lock. Gas volumes are expressed as dry gas at 0°C, assuming a constant pressure of 1 atm.

### Statistics

All statistical analyses were performed using one-way analysis of variance (ANOVA) and Tukey's multiple comparison test using the statistical software Prism (Prism 5 for Mac OS X, version 5.0b; GraphPad Software Inc., La Jolla, CA, USA). The term 'significant' is only used where a statistical analysis of significance has been performed. The significance level of 5% was used throughout all statistical analyses. Values are given ± 1 SD. The SDs of weight losses during ensiling, of TS and VS determinations, of the concentrations of volatile compounds added to the corrected values of TS and VS and of tests and controls in BMP were combined according to standard statistical rules [33] to provide a SD of the final result. For linear combinations (Equation 3) the SDs were combined according to Equation 4 [33]. For multiplicative expression (Equation 5) the SDs were combined according to Equation 6 [33]:

(3) $$\text{y}=\text{k}+{\text{k}}_{\text{a}}\text{a}+{\text{k}}_{\text{b}}\text{b}+{\text{k}}_{\text{c}}\text{c}+...$$

(4) $$\sigma_{\text{y}}=\surd \left({\left({\text{k}}_{\text{a}}\sigma_{\text{a}}\right)}^{2}\;+{\left({\text{k}}_{\text{b}}\sigma_{\text{b}}\right)}^{2}+{\left({\text{k}}_{\text{c}}\sigma_{\text{c}}\right)}^{2}+\ldots \right)$$

(5) y=kab/cd

(6) $$\sigma_{\text{y}}∕\text{y}=\surd \left({\left(\sigma_{\text{a}}∕\text{a}\right)}^{\text{2}}+\;{\left(\sigma_{\text{b}}∕\text{b}\right)}^{\text{2}}+\;{\left(\sigma_{\text{c}}∕\text{c}\right)}^{\text{2}}+\;{\left(\sigma_{\text{d}}∕\text{d}\right)}^{\text{2}}+\ldots \right)$$

Where σ = standard deviation; a, b, c, d = independent measured quantities; and k = constant.

## Competing interests

The authors declare that they have no competing interests.

## Authors' contributions

LB became aware of the methodological problem investigated, and secured financial support for this study. All authors participated in the design of the study, harvesting of the crops and reviewing of the literature. EK set up the ensiling method and performed most of the ensiling experiments, the TS and VS determinations and all analyses of the volatile compounds. IAN participated in the ensiling trials and carried out the BMP tests. EK performed the statistical analysis and prepared the major part of the manuscript. LB and IAN contributed to writing the manuscript, and all authors read and approved the final manuscript.
